# Illinois—Dr. Swain’s Letter

**Published:** 1901-05

**Authors:** 


### ILLINOIS—DR. SWAIN'S LETTER.

THE JOURNAL is ever willing to loan its columns for the publication of all matters of interest to the profession; to encourage the free discussion of all subjects of importance to the profession, and to aid in the settlement of all questions that involve the welfare of the profession. Our position in regard to the legislation in Illinois in establishing a State Board of Veterinary Medical Examiners under the auspices of the State Live-stock Commissioners was well known at the time as against the wisdom of this plan. Its outcome in the

State has divided the ranks of the profession. There is much to be said for and against both sides in the wordy conflict now going on, but we believe the greater danger is to the profession as a whole, and we will not publish any further letters on the subject which are so personal in character. We strongly urge that the profession get together and endeavor to correct the evils complained of.

